# Numerical format integration in primary school children examined with frequency-tagged electroencephalography

**DOI:** 10.1038/s41598-025-11281-7

**Published:** 2025-08-05

**Authors:** Mila Marinova, Christine Schiltz

**Affiliations:** 1 Institute of Cognitive Science and Assessment, Department of Behavioural and Cognitive Sciences, Faculty of Humanities, Education and Social Sciences, University of Luxembourg, Maison des Sciences Humaines 11, Porte des Sciences, Belval, Esch-sur-Alzette, L-4366 Luxembourg; 2 Brain and Cognition, KU Leuven, Leuven, Belgium; 3 Faculty of Psychology and Educational Sciences, KU Leuven @Kulak, Kortrijk, Belgium

**Keywords:** Cognitive neuroscience, Human behaviour

## Abstract

Mastering the relationship between different numerical formats (i.e., digits, number words, and non-symbolic quantities) is an important foundational skill for later math competencies. However, the neurocognitive mechanisms of this relationship remain poorly understood in children. Consequently, the current study examines the integration between digits, number words, and dots in a sample of 34 primary school children (aged 7 to 14 years) with an electroencephalography paradigm tagging the frequency of stimulus presentation. In an oddball paradigm, we presented children with mixed notation sequences (i.e., dots – words, digits – dots, words – digits) at a rate of 166 ms per stimulus while manipulating the magnitude of the deviant numbers in an experimental (rule = standards < 5, deviants > 5) and control conditions (no rule). We observed deviant responses in the experimental but not in the control condition, with the strongest responses for dots – words, followed by words – digits and finally, digits – dots. These findings suggest that, in children, numerical magnitude information is automatically extracted irrespective of the format at least under implicit and rapid presentation conditions.

**Supplementary Information:**

The online version contains supplementary material available at 10.1038/s41598-025-11281-7.

## Introduction

To acquire advanced mathematical competencies, children must learn to map culturally acquired symbolic numbers – such as Arabic numerals (e.g., “6”) and number words (e.g., “six”) – onto evolutionarily ancient, non-symbolic quantity representations, typically expressed as arrays of objects (e.g., “●●●●●●”;^1^). A central focus in numerical cognition research is understanding how these distinct numerical formats are integrated. Some accounts propose that the processing of symbolic numbers entails the automatic activation of their corresponding approximate, non-symbolic representations (i.e., “20” → “●●●●●●…”^2–4^). In contrast, other views suggest that symbolic and non-symbolic systems are largely distinct and interact only under specific conditions^5^.

A related, but distinct question is how this relationship between symbolic and non-symbolic formats evolves across development. Some researchers argue that the core number system supports the acquisition of small symbolic numbers (i.e., “one”/“1” →“●”, “two”/“2” → “●●”, etc.) up to around five, after which symbolic knowledge becomes increasingly independent^6–9^. According to this view, the two systems gradually estrange with age and experience^10^. However, the precise neurocognitive mechanisms underlying the relationship between symbolic and non-symbolic numbers in children remain poorly understood, particularly because most existing studies examine only two numerical formats, typically digits and dots (for review see^11^), and few include all three (e.g., dots, digits, and number words) in a single paradigm^12,13^.

In the current study, we recorded electroencephalography (EEG) responses tagged at the frequency of the stimulus presentation to shed further light on the neurocognitive development of numerical cross-format processing. Primary school children (aged 7 to 14) viewed cross-format number sequences (digits – words, words – dots, dots – digits) in an oddball design (standard stimuli < 5, deviant > 5) in an implicit task setting. This way, we probed whether children automatically integrated the numerical formats while avoiding potential confounds from task-related decisional strategies.

### Mapping between digits, number words, and dots

It is widely accepted that humans possess two core systems for representing numerical information—often referred to collectively as the “number sense”^14^. The first, the Approximate Number System (ANS), encodes numerical magnitude imprecisely but has an unlimited capacity (e.g., “●●●●●●…” ≈ [iiiiii…]). The second, the Object Tracking System (OTS; also known as Parallel Individuation, PI), allows for the precise representation of small quantities (e.g., “●●●” = [i][j][k]), but is limited to sets of about 4–5 items.

While most developmental models in numerical cognition agree that these core systems support the acquisition of symbolic number knowledge^15–18^, they diverge in terms of the mechanisms involved and the specific role of each system. One line of research argues that symbolic numbers are learned via direct mappings onto the ANS, and that such mappings persist even for relatively large symbolic values in adults (e.g., > 15^19^). Supporting this claim, studies show that young children’s performance on numerical mapping tasks involving small numbers (1–6) varies systematically across format pairs: highest accuracy is typically found for words–dots, followed by dots–digits, and lowest for digits–words (Benoit et al., 2013). This has been interpreted as evidence that children initially map symbolic formats onto core representations and only later derive associations between symbolic formats themselves (e.g., “2” = “●●” → “two”). In contrast, other researchers propose that symbolic number knowledge builds initially on the OTS for learning small number words, but that acquisition of larger symbolic numbers (i.e., > 5) relies on semantic bootstrapping processes. In this view, the associations among symbolic representations become the primary mechanisms of further number learning^5,6,20^. For instance, Marinova et al.^21^ tested 195 kindergarten children aged between 2y6m and 5y2m and observed that children first learn to map between small (1 to 4) number words and dots, followed by number words and digits, and only after between dots and digits. Additionally, mediation analysis showed the best result when considering that the words – digits mapping performance predicted the relationship between dots – words and digits – dots. Together with previous findings^22,23^, these results suggest that when learning to map between dots and digits, children rely on their knowledge of how the two symbolic number formats (i.e. number words and digits) relate to each other and how symbolic number relates to non-symbolic quantities (i.e., “two” = “●●”, and “two” = “2”, then “2” → “●●”). These findings emphasize that core numerical representations are not the sole driving mechanisms behind the early mapping abilities and that their contribution, if any, might be limited to small quantities^8,24,25^.

Further evidence from primary school-aged children (6 to 11 years old) corroborates the findings in kindergarten children by demonstrating that the relationship between the symbolic and non-symbolic number representation is not as intuitive as previously thought, and the two representations may follow different developmental trajectories^13,26,27^. For instance, in a study by Marinova and Reynvoet^27^ primary school children performed an audio-visual number comparison in four conditions (number range 4–28): spoken number words – visually presented digits (i.e., purely symbolic), auditory presented tone sequence – visually presented dots (i.e., purely non-symbolic), number words – dots and tones – digits (i.e., mixed format). The results showed a ratio effect – that is a more efficient performance when the relative distance between two numbers is large (5 vs. 8) compared to small (7 vs. 8), in all conditions containing at least one non-symbolic stimulus (i.e., tones – digits, tones – dots, number words - dots), but not in the purely symbolic condition (i.e., number words - digits). Because the ratio effect is considered as an index of the underlying approximate representations, the authors concluded that its absence in the purely symbolic condition suggests that, already in children, a distinct cognitive system underlies the processing of symbolic numbers. Overall, although theories of numerical cognition emphasize that core numerical representations play a role during the acquisition of symbolic numbers, there is a growing body of research challenging the assumption that symbolic and non-symbolic numbers are automatically mapped onto each other in children.

Only a handful of studies have examined the cross-format mappings in children (e.g^13,28^). In a 2021 study, van Hoogmoed et al.^13^ examined the relationship between symbolic and non-symbolic numbers in a sample of older children (9 to 12 year-olds) using electroencephalography (EEG) and an event-related potentials methodology (ERP). In this study, 34 children performed a match-to-sample task (press a response key only when the prime and the target are the same in terms of their magnitude) in purely non-symbolic (dots – dots), purely symbolic (number words –digits) and mixed conditions (digits – dots, dots – digits). The number range varied between 20 and 40. The authors hypothesized that if the non-symbolic numerosities are automatically associated with the symbolic numbers one should observe a format-independent ratio effect and a similar ERP pattern for dots across conditions (i.e., regardless of whether they are primes or targets).

For the dots–dots condition, the results showed a late (after 650 ms) significant ratio effect within the parietal region in combination with early (200 ms) occipital responses to the non-numerical visual cues (i.e., surface and diameter). Interestingly, the symbolic task also had a ratio effect, but with a different pattern: early (50–300 ms) central and later left frontocentral responses (700–800 ms). Finally, comparison across conditions showed that the ERP responses for dots as primes did not differ across conditions, but the responses for the dots as targets differed, suggesting that the prime format (dots or digits) did influence the subsequent processing of the dots. Because the overall pattern of results varied across the numerical formats, the authors suggested that the symbolic and non-symbolic numbers are processed in separate cognitive systems and indicating their estrangement.

Yet, certain limitations of this study warrant further investigations. For instance, the authors used a match-to-sample task, requiring the participants to respond when the prime and target are of the same value. Although only the non-match trials were analysed in their study, the task is still explicit since the participants should actively process the magnitude or numbers. Indeed, earlier work by Cohen Kadosh and Walsh^29^ suggests that using implicit task instruction would allow probing the numerical representations, and presumably their relationship, per se and free of any decisional strategies. Additionally, behavioural and neurocognitive work in adults^30,31^ suggests that evidence for estrangement is more likely to occur under explicit task settings (e.g., magnitude comparison task), while integration is observed under implicit task settings (e.g., number – letter identification task), thus confirming earlier suggestions that the task instructions may influence whether the numerical format are processed in a format-(in)dependent manner in the brain.

Furthermore, van Hoogmoed et al.^13^ only included number words in their design as primes. Given the perceptual differences across conditions, analysing the associated ERP responses was impossible, thus leaving the question about the neurocognitive underpinnings of all three numerical formats (digits, words, and dots) open in children. Additionally, interpretation based on responses for larger number ranges (e.g., above 10) proves challenging across formats. On the one hand, double-digit numbers might require a decomposing strategy, as previous work suggests^32^. On the other, in languages such as Dutch or German, there is an inversion of the double-digit numbers (i.e., “three and twenty” instead of “twenty-three”), thus rendering a cross-format condition such as visually presented digits and number words (e.g., “23” vs. “five and twenty”) very different in cognitive and visual terms compared to dots – words or dots – digits condition, where it is unlikely that participants will have to do a decomposition and inversion at the same time to compare the numbers^13^. Consequently, single digits might be a better option if we want to test all three numerical formats.

To overcome these issues one could examine the relationship between the numerical formats using fast visual presentation (e.g., 100 ms per stimulus) and under implicit task settings, which would encourage automatic processing. An emerging EEG methodology in numerical cognition, referred to as fast periodic visual presentation or frequency–tagged EEG – seems promising. Frequency-tagging has already been successfully employed to study within and cross-format number processing in various adult populations^33–37^, and to study lateralisation patterns for digits and letters as well as cross-format number processing in canonical dot (e.g., dice patterns) and finger configurations in young children aged 6 to 10 years^28,38^. We discuss this methodology in more detail below.

### Frequency-tagged EEG

The fundamental idea behind frequency-tagged EEG is similar to the well-known technique of steady-state visually evoked potentials (SSVEP)^39–41^. Concretely, if a stimulus (e.g., word) is presented at a particular frequency (e.g., F = 10 Hz or 100 ms per stimulus), it leads to a visual response at the frequency of presentation and its harmonics (2f, 3f, etc.). The oddball version of the SSVEP consists of the periodic presentation of two categorically different stimuli (for other paradigms, see^40^). For example, suppose standard category stimuli (e.g., words) appear at a fast rate of F = 10 Hz, with every n^th^ item (e.g., every 8th stimulus) being a deviant (e.g., non-word). In that case, the presentation sequences look like this: W-W-W-W-W-W-W-NW-W-W…If the system automatically discriminates between the standard and the deviant categories (W vs. NW), we should observe a selective response at the deviant frequency *F/n* (i.e., 1.2 Hz) and its harmonics (i.e., 2.4 Hz, 3.6 Hz, etc.).

In a previous study, we used this paradigm to examine within and cross-format processing of digits, number words, and dots in adults^34,35^. Concretely, in the study by Marinova et al.,^34^, participants saw mixed format (digits – words, words – dots, and dots – digits) in an oddball design. Stimuli were presented at a fast rate of 10 Hz (i.e., 100 ms per item) and a deviant was inserted for every 8th item (i.e., at 1.25 Hz). In the experimental condition, the standard stimuli were numbers smaller than 5, and the deviants were numbers larger than 5. In the control condition, small and large numbers were randomly intermixed. The authors hypothesized that significant oddball responses in the experimental condition would indicate that adults automatically discriminate between small and large numbers, regardless of their format. Indeed, results showed evidence for automatic cross-format integration in the digits – words and dots – words condition, but not in the digits – dots condition. According to the authors, the results suggest that a direct cognitive link exists between the dots and number words and between the digits and number words, which can be observed early in the visual stream, while the link between dots and digits is probably indirect and occurs at a later processing stage. Later, Marlair et al.^35^ corroborated these findings by showing a cross-format distance effect in adults. Concretely, in this study, the authors stimulated participants at the 6/1.2hz rate and presented them mixed sequences of numerical representations (digits, words, canonical dot configurations and canonical finger configurations) while manipulating the numerical distance between the standard and the deviant (small numerical distance: standard = 2/ deviant = 3, and large numerical distance: standard = 2/deviant = 8). The results showed stronger right-lateralized oddball responses when the distance between the standard and the deviant was large, compared to when the distance was small. Overall, evidence from frequency-tagged EEG studies shows that adult participants automatically integrate magnitude information across formats, except in digits – dots conditions. However, the developmental perspective of the automaticity of this integration remains yet to be examined, as corresponding frequency-tagged EEG studies in younger populations are still missing.

### The current study

Based on the findings and the experimental design of Marinova, et al.^34^, the current study examined cross-format integration of numerical information in primary school children using frequency-tagged EEG. More precisely, we used an oddball design with a stimulation rate of 6/1.2 Hz (i.e., 166 ms per stimulus, every 5th is a deviant) and presented children with mixed sequences of digits, words, and dots. In the experimental condition, the standard stimuli were numbers smaller than 5, while the deviants were numbers larger than 5. In the control condition the small and large category were randomly intermixed. In the current study, we used relatively small numbers (i.e., 1 to 9) because numbers within this range tend to be represented with a higher precision than larger numbers (i.e., > 15^42^), allowing us to compare the performance across conditions. We hypothesized that if children automatically integrate numerical magnitude, independently of the format, we should observe significant oddball responses in the experimental condition in response to the magnitude change but not in the control condition lacking such a magnitude-related difference between standard and deviant stimuli (H1). Alternatively, a lack of significant oddball responses would indicate that numerical magnitude across formats is not processed automatically in primary-school children (H0). Furthermore, based on previous developmental studies^13,21–24^, we expected that the oddball responses would be strongest in the digits – words and words – dots, and weaker, if present, in the dots – digits (H2a). Based on integration studies with adults (e.g^34^), we also expected an interaction between the sequence and the electrode site, suggesting different topographies (H2b): left lateralised for words – digits, and left occipital for dots – words, and right occipital for dots – digits (if present).

## Methods

### Participants

The sample consisted of 34 primary school children aged between 7 years 4 months and 14 years 4 months (M_*age*_ = 124.94 months (i.e., 10 years 5 months), SD = 19.80 months (i.e., 1 year 8 months), 12 females and 22 males). All children had normal or corrected-to-normal vision, no functional impairments, and followed a Luxembourgish school curriculum. All participants were multilingual (i.e., they spoke more than two languages). With respect to the language of instruction and the language of testing (i.e., German), 67.65% were L2-speakers (i.e., they spoke a language different than German or Luxembourgish with at least one of their parents/legal representatives), and 32.35% were L1 speakers (i.e., they spoke Luxembourgish or German with both their parents/legal representatives). A total of 79.41% of the children said that they preferred to count in German and/or Luxembourgish, 14.71% preferred Portuguese, and the final 5.88% preferred other languages. It is worth noting that although this sample seems unconventionally heterogeneous, it is representative of the Luxembourgish population, which is very diverse linguistically and culturally^43,44^. In addition, in the local educational context, it is not uncommon for some 13- or 14-year-olds (*N* = 2) to remain in primary school, particularly in cases of grade repetition or delayed school entry.

The Ethical Review Panel at the University of Luxembourg approved the experimental protocol for the current study (ERP 21–045). Approval for testing in public schools was obtained from Luxembourg’s Ministry of Education. All methods were performed in accordance with the relevant guidelines and regulations, including the Declaration of Helsinki and its latest amendments. Nineteen participants were tested at their primary school and 15 were tested at the cognitive neuroscience lab of the University of Luxembourg. Prior to participation, the parents/legal representatives received a detailed information sheet describing the procedure as well as the exclusion criteria (i.e., history of epilepsy, head trauma, learning and/or attentional difficulties). Only children whose parents/legal representatives signed the informed consent were tested.

### Tasks and procedure

Each child completed three experimental tasks, performed in the order described below. The testing sessions lasted approximately 50 to 75 min per child with regular breaks. All tasks and instructions were executed in German by a native German/Luxembourgish-speaking experimenter.

#### Socio-demographic questionnaire

The questionnaire was completed by the child with the help of the experimenter and contained questions about general demographic information (i.e., biological sex and date of birth), the child’s language profile (i.e., which languages are spoken with the mother and with the father), language preferences (i.e., in which language(s) does the children prefer to speak and in which language(s) does the child prefer to count), as well screening questions for exclusion criteria (i.e., history of epilepsy; presence of any learning or attention difficulties diagnosed by qualifies mental health professional).

#### Speeded number words reading task

This task was used as a pre-screening measure to ensure that the child could read the number words presented on the screen at the speed of the periodic visual stimulation: 166 ms per stimulus. Concretely, children were presented with number words from “one” to “nine” in German and were asked to say out loud which number word they saw. Each trial started with a 500 ms fixation cross, followed by a number word presented for 166 ms. This was followed by a blank screen, which stayed until response. After 1500 ms intertrial interval, the next trial started. Children could respond verbally during the stimulus presentation and/or during the presentation of the blank screen. The responses (correct or incorrect) were noted in a separate sheet by the experimenter. After the response was recorded, the experimenter pressed “SPACE” for the next trial. Each number word appeared 3 times for a total of 27 randomly presented trials. There were no training trials. PsychoPy software^45^ was used for stimulus presentation. The task took approximately 2–3 min and children received positive feedback regardless of their performance. All children performed at 100% accuracy, suggesting that the sample comprised of proficient German speakers.

#### Fast periodic visual stimulation (FPVS) with EEG

**Fig. 1:**
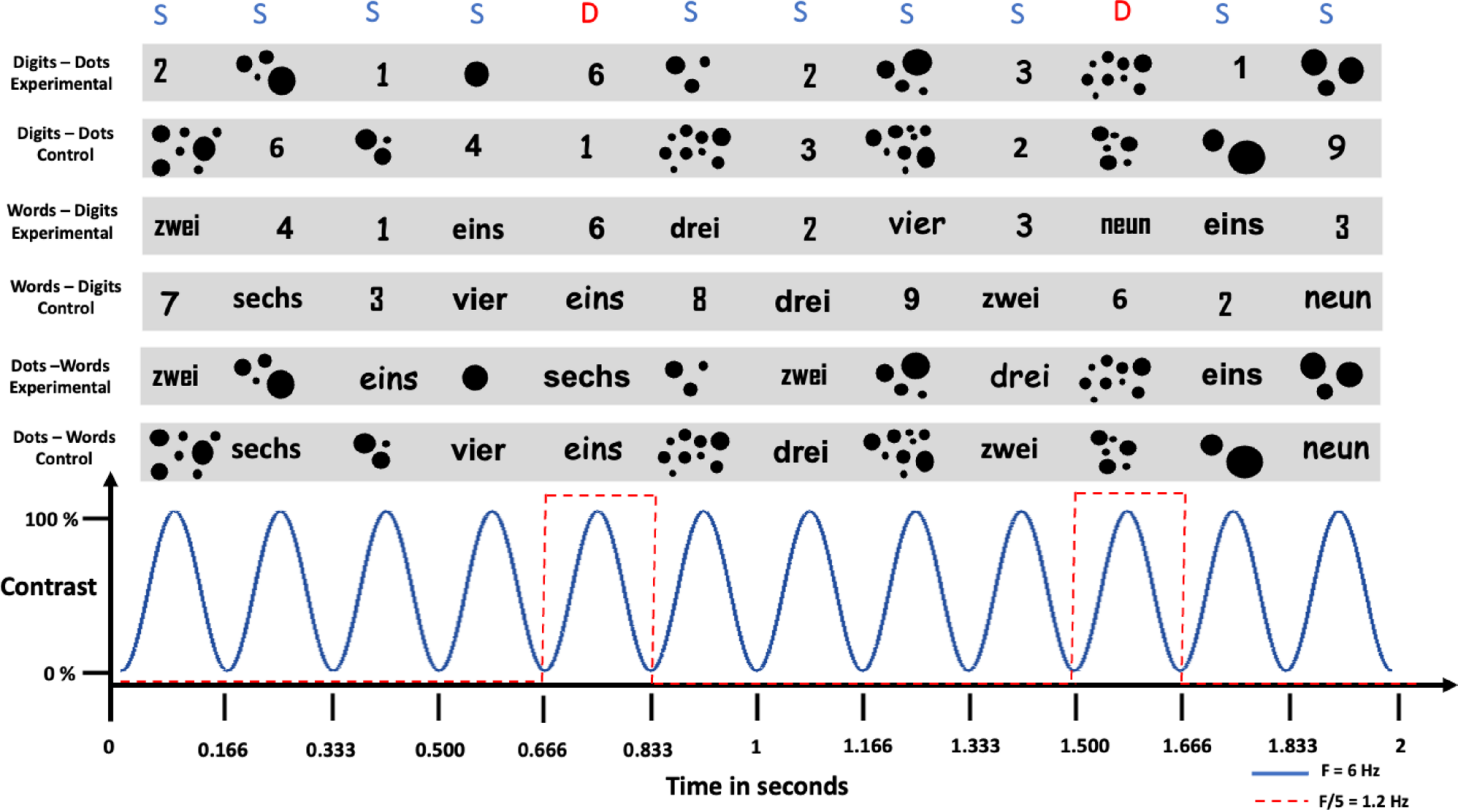
Visual Depiction of the of the study’s design. Sequences were presented for 44 s in both experimental and control conditions. For the experimental conditions, participants saw mixed notation sequences (digits – dots, words – digits and dots – words). Irrespectively of the notation, numbers smaller than five were presented as standard stimuli (S) at the frequency of 6 Hz, and every 5th item, a number larger than 5 was inserted at the deviant (D) frequency of 1.2 Hz. In the corresponding control conditions, the categories (standard vs. deviant) were assigned randomly. The bottom panel illustrates the onset and the offset of each stimulus following the sinusoidal contrast modulation from a 0 to 100% contrast. The number words were presented in German, which translated into English as follows: “eins”—“one”, “zwei”—“two”, “drei”—“three”, “vier”—“four”, “sechs”—“six”, “sieben”—“seven”, “acht”—“eight”, “neun”—“nine”. This is conceptual, not an actual depiction of the stimuli’s appearance and their physical properties.

The task employed FPVS oddball design with a presentation rate of 6 Hz/1.2 Hz for the standard and deviant stimuli, respectively. Put differently, the stimuli were presented at a fast rate of 6 Hz (i.e., 6 stimuli per second or 166.66 ms per stimulus), with every 5th stimulus being a deviant (i.e., 1.2 Hz). The stimuli were depicted as digits, words, or dot configurations, thus giving raise to the following mixed-format conditions: Words–Digits, Dots–Words, and Digits–Dot (Fig. 1). The stimuli were presented as black symbols on grey background in a picture format. The final pictures were rescaled to 0.7 or their original size and presented centrally using 800 × 600 px resolution, while participants were seated conformably 1 m from the screen (LED monitor 540 × 330 mm). Consequently, the size for the digit stimuli, on average, varied between 21 × 45 px (i.e., 0.81 × 1.42 degrees of visual angle) and 30 × 48 px (i.e., 1.16 × 1.51 degrees of visual angle). The size of the words, on average, varied between 109 × 49 px (i.e., 4.21 × 1.54 degrees of visual angle) and 174 × 50 px (i.e., 6.72 × 1.58 degrees of visual angle). Finally, the random dots configurations were presented as pictures with size of 216.3 × 216.3 px (i.e., 8.35 × 6.80 degrees of visual angle). To control for perceptual habituation to the visual properties of the stimuli, the dot configurations as well as the fonts of the digits and words varied. For the dot stimuli, there were five different dot configurations per magnitude. The dot stimuli were generated with the MATLAB script by Gebuis and Reynvoet^46^, controlling for non-numerical cues (i.e., total surface, convex hull, density, dot size and circumference). For the words and digits stimuli, each stimulus was foreseen to appear in five different fonts (Arial, Agency FB, Comic sans MS, OCR A extended, Lucida sans). However, due to technical error, the stimuli were depicted only in four fonts (i.e., all but Lucida sans).

For each mixed-format condition, there was an experimental and control version. In the experimental condition, the standard category consisted of numbers smaller than 5 (i.e., 1,2,3,4), while the deviant category consisted of numbers larger than 5 (i.e., 6,7,8,9). The notation of the standard and deviant stimuli randomly varied depending on the format condition. For example, in the word-digits condition the standard stimuli contained both words and digits. Similarly, the deviant stimulus randomly appeared as a word or a digit. In the control condition, there was no rule, meaning that small and large numbers (in their various notations) were randomly intermixed. Each sequence in a condition started with 2 s fade-in phase, followed by 40 s of stimulation phase, after which 2 s fade-out followed, resulting in a 44s per sequence. Each condition contained 4 sequences, resulting in a total of 24 sequences per participant. The presentation was blocked per notation and per condition. For example, participants saw all four repetitions of the words – digits experimental condition, before moving to the control condition. The order of the notations as well as the conditions within the notations, were counterbalanced in a Latin square design. To maintain constant attention, participants were asked to fixate at the center of the screen, where a fixation cross was continuously presented. Their task was to press the space bar once the color of the cross changed from blue to red. The color changed occurred 8 times per sequence at random intervals.

#### EEG data acquisition and preprocessing

Participants were seated comfortably 1 m from the screen. The EEG signal was acquired at 2048 Hz using a 64-channel Biosemi ActiveTwo system (Biosemi, Amsterdam, The Netherlands) with the electrode location according to the standard 10–20 system (for details see http://www.biosemi.com). The Common Mode Sense (CMS) and the Driven Right Leg (DRL) served as the reference electrodes. The electrode offset was held below 40 microvolts (µV). To decrease the processing load, recordings were resampled from 2048 to 512 Hz. Further data processing steps and analyses were done using Letswave 6 software (https://nocions.github.io/letswave6/). Data were filtered (band-pass cut off 0.1–100 Hz) and segmented into a 44 s sequences for visualisation and detecting artefact/noise. Noisy channels were interpolated using three of the closest neighbouring electrodes (no more than three electrodes, i.e., 5% of the electrodes per participant). Individual repetitions, where the EEG signal was too noisy to correct were removed. This resulted in the following total number of repetitions per condition (experimental vs. control): Words – Digits: 127 vs. 120 reps, Dots – Words: 128 vs. 121 reps, and Digits – Dots: 152 vs. 111 reps (It is worth noting that preliminary analyses of the data (including the noisy repetitions) yielded the same overall findings). Afterwards, the EEG signal was re-referenced to the common average and segmented in 40 s epochs (i.e., excluding 2 s fade-in and 2 s fade-out) containing 400 stimulus onsets and 20,480 data points. Then, we averaged the repetitions of each condition for each participant and applied Fast Fourier Transformation (FFT). Three regions of interests were defined a-priori based on previous studies^34,37,38^: Medial Occipital (MO: O1, Iz, Oz, O2), Left Occipito-Parietal (LOP: P5, P7, P9, PO7), and finally Right Occipito-Parietal (ROP: P6, P8, P10, PO8). As can be seen on Fig. 3, these a-priori ROI were compatible with the topographies obtained in the current study.

#### EEG data analysis

**Fig. 2:**
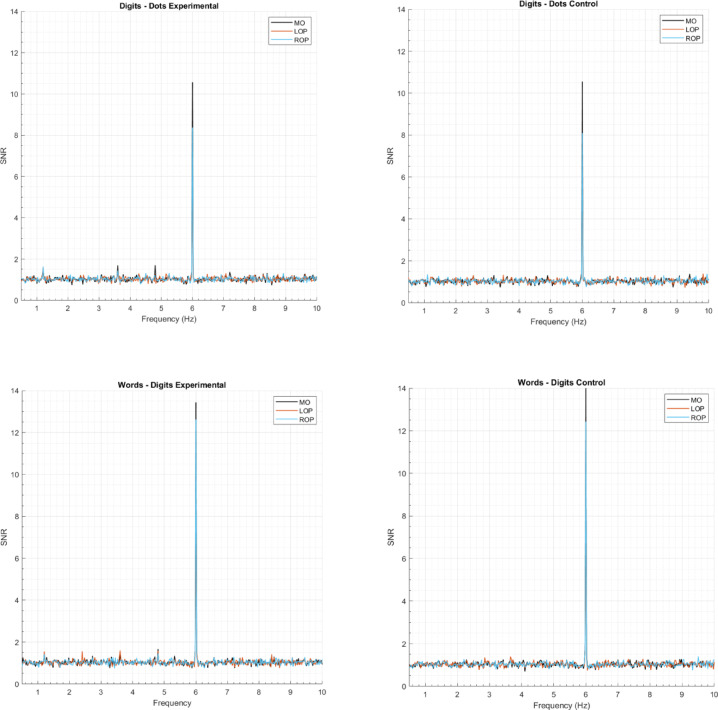

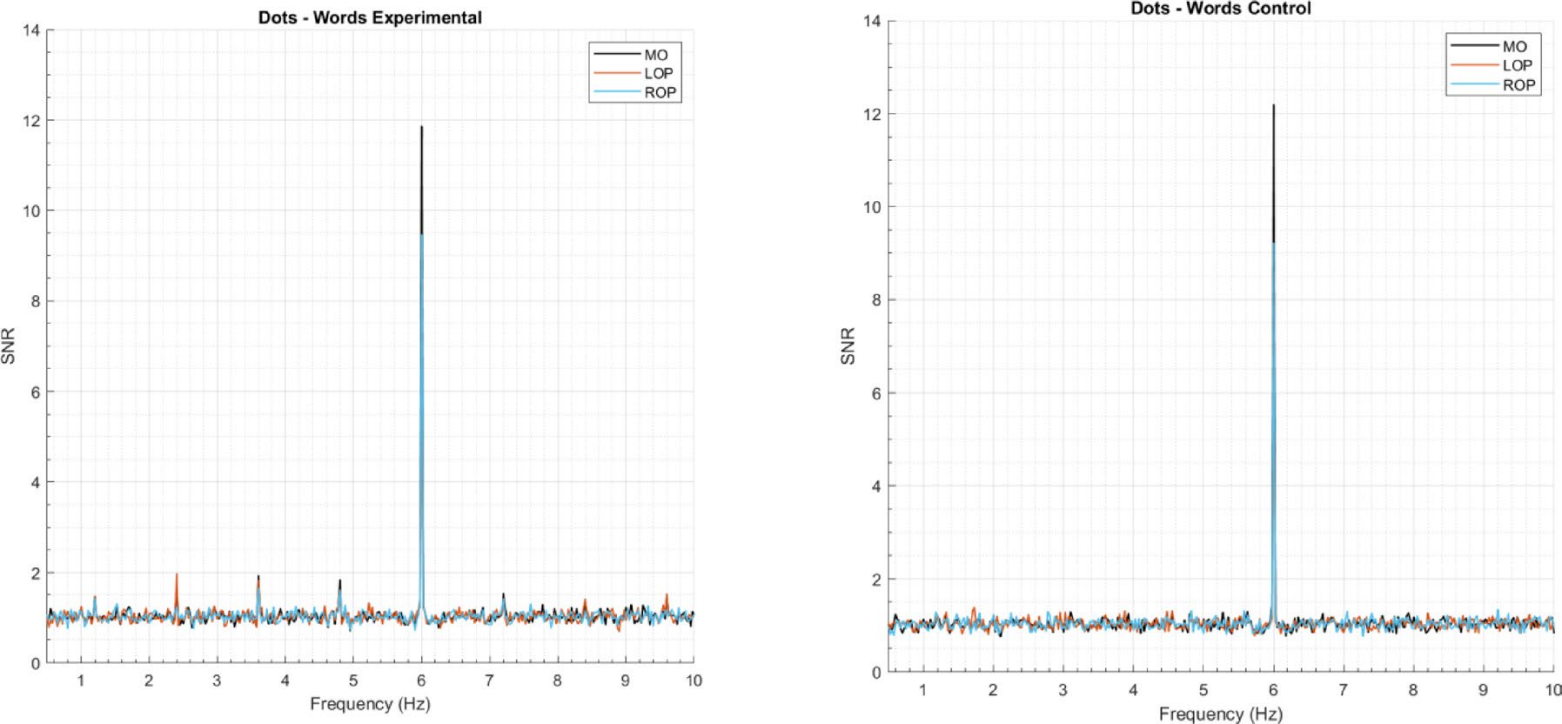
Amplitude spectra expressed as SNR (signal-to-noise ratio) of the standard frequency (6 Hz) and the deviant frequency (1.2 Hz) and its harmonics, depicted per notation, condition, and ROI.

**Fig. 3:**
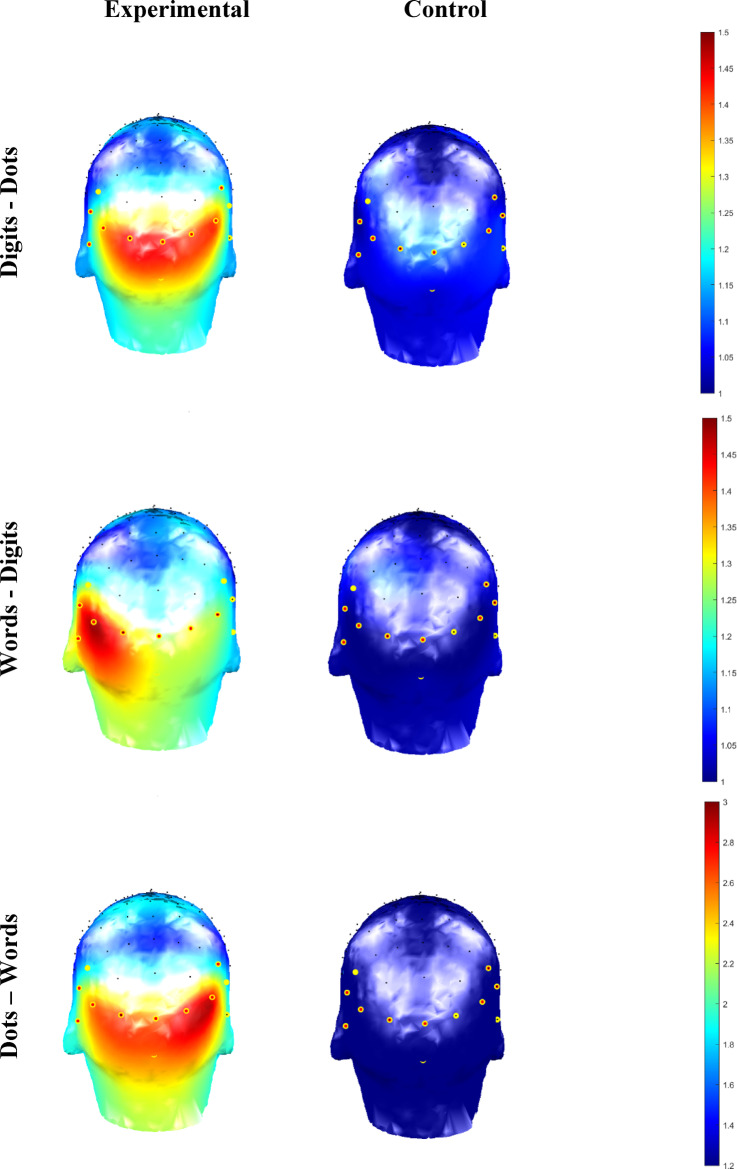
Scalp topographies of the deviant response (1.2 Hz) and its six harmonics (1.2 Hz. 2.4 Hz., 3.6 Hz, 4.8 Hz, 7.2 Hz, and 8.4 Hz) expressed as SNR and depicted per notation and condition. Selected ROI are highlighted in yellow.

First, to estimate the Signal-to-Noise Ratio (SNR) across the spectrum, each frequency was divided by the mean amplitude of the twenty surrounding bins (10 on each side) to each bin, excluding the immediately adjacent bins and two most extreme bins^38,39,47,48^. We then averaged the data across participants and per notation condition. The amplitude spectra expressed as SNR for each notation condition is depicted in Fig. 2. Based on this visual inspection, deviant responses were present up to the 6th harmonic, excluding the standard frequency (i.e., 1.2 Hz, 2.4 Hz, 3.6 Hz, 4.8 Hz, 7.2 Hz, and 8.4 Hz), while the amplitude for the standard frequency was strongest at the first harmonic (i.e., 6 Hz). Figure 3 depicts the mean response for the deviant frequency and its 6 harmonics, expressed as SNR for each notation and condition.

Second, to assess the statistical significance of the oddball responses, we used z-scores^38,39,47,48^. These z-scores were obtained by first segmenting the individual FFT data around the response of interest and its harmonics (e.g., 1.2 Hz, 2.4 Hz, 3.6 Hz, 4.8 Hz, 7.2 Hz, and 8.4 Hz) into successive bins (12 on each side) and then summing across the frequency spectra. The same procedure was applied to the standard responses (i.e., 6 Hz). After that, we applied z-score transformations to each bin as a function of its twenty surrounding bins (10 on each side). Conventionally, z-scores above 1.64 (i.e., *p* < .05 one-tailed; hypothesis tested signal < noise) indicate significant brain responses to the deviant category. We applied the same procedure to examine the general visual response at the standard stimulation frequency (i.e., 6 Hz). However, based on visual inspection, we limited the analyses only to the first harmonic since that one yielded the highest amplitude.

## Results

The study’s data set and statistical output are freely available on the Open Science Framework (https://osf.io/ktm94/).

Table 1 depicts the z-scores for both standard and deviant responses per Notation, Condition, and Region of Interest (ROI). The z-scores for the general visual responses (i.e., at the standard frequency of presentation 6 Hz) were all significant, confirming that participants attended to the stream of visual stimuli. The results concerning the oddball responses demonstrated first that all experimental conditions yielded stronger responses than the control conditions. Second, significant deviant responses were present for all mixed notations, but not across all ROIs. Concretely, the deviant responses were significant only in the Medial Occipital region (MO) for the Digits – Dots condition. Responses were present only in the Left Occipito-Parietal (LOP) region for Words – Digits. Finally, significant responses were present for Dots – Words in Right Occipito-Parietal (ROP) and MO.

**Table 1:** Averaged z-scores for the standard and the deviant responses (with the corresponding standard error (SE) of the mean), depicted per condition, notation, and ROI. Values > 1.64 are considered significant (in bold). Control conditions are in italic.

			Region of Interest (ROI)		
Notation	Amplitude	Condition	ROP	LOP	MO
Digits – Dots	Standard	Experimental	**14.37 (1.18)**	**10.47 (1.23)**	**18.39 (1.20)**
		*Control*	***14.00 (1.21)***	***9.66 (1.20)***	***18.08 (1.39)***
	Deviant	Experimental	1.25 (0.19)	1.17 (0.25)	**1.86 (0.27)**
		*Control*	*0.25 (0.19)*	*0.17 (0.18)*	*0.28 (0.20)*
Words – Digits	Standard	Experimental	**20.48 (1.52)**	**14.77 (1.21)**	**22.17 (1.54)**
		*Control*	***20.69 (1.66)***	***15.50 (1.19)***	***24.04 (1.53)***
	Deviant	Experimental	0.99 (0.27)	**2.07 (0.33)**	1.25 (1.35)
		*Control*	*-0.09 (0.17)*	*-0.08 (0.18)*	*-0.21 (0.18)*
Dots – Words	Standard	Experimental	**15.89 (1.17)**	**14.19 (1.27)**	**19.69 (1.19)**
		*Control*	***15.75 (1.25)***	***14.19 (1.23)***	***20.39 (1.33)***
	Deviant	Experimental	1.58 (0.18)	**2.56 (0.30)**	**2.43 (0.24)**
		*Control*	*0.07 (0.19)*	*0.41 (0.20)*	*0.49 (0.21)*

To examine the standard and the deviant responses further, we conducted a repeated measures ANCOVA with Notation (3 levels: digits – dots, words – digits, dots – words), Condition (2 levels: experimental and control), and ROI (3 levels: ROP, LOP, and MO) as within-subject factors and age (in months) as a covariate. Data were analysed in classical and in Bayesian statistical frameworks. We report the Bayes factors (BF) - the ratio of the likelihood of the alternative hypothesis and the likelihood of the null hypothesis. Conventionally, the evidence provided by the BF values is categorised as “anecdotal” (for values between < 1 and 3), “moderate” (for values between 3 and 10), “strong” (or values between 10 and 30), “very strong” (for values between 30 and 100), and “extreme” (for values > 100). Given that the Bayesian framework allows us to quantify the evidence for both alternative and null hypotheses, we opted to report the Bayesian analyses. To obtain both classical and Bayesian results, we used JASP statistical package v 0.18.1.0 (https://jasp-stats.org/). Finally, the analyses reported here were run with the program’s default priors (i.e., r cale fixed effects = 0.5, random effects = 1, covariate = 0.354). The statistical output of using narrower (i.e., ½ lower than the default) and wider priors (i.e., ½ higher than the default) and using the classical statistical framework were similar to the ones reported here and are available on the OSF (https://osf.io/ktm94/).

### Response to the standard frequency (6 Hz)

*Assumption checks for the standard responses (i.e.*,* general visual response)* showed that the z-scores per Condition, as well as the Age, were all normally distributed (Shapiro-Wilk *p* > .05), apart from two conditions: digits – dots and dots – words, where the z-score for the experimental conditions in the Right and Left Occipito-Parietal ROIs showed slight deviations from normality (*Skewness*_digits – dots exp ROP_ = 0.76, SE = 0.40, *Kurtosis*_digits – dots exp ROP_ = -0.26, SE = 0.79, *p* = .03 *Skewness*_dots – words exp LOP_ = 0.96, SE = 0.40, *Kurtosis*_dots – words exp LOP_ = 0.78, SE = 0.79, *p* = .03). Visual inspection of the Q-Q plots (see Fig. 1S in Supplementary) showed that the residuals are mostly normally distributed. Mauchly’s test indicated that the sphericity assumption was violated for the interaction between Notation and ROI, *p* = .03, Greenhouse-Geisser correction was applied for the frequentist analyses. However, for the Bayesian analyses, sphericity corrections are not readily implemented^49^. Consequently, we cautiously approach any results where this assumption is violated.

**Fig. 4:**
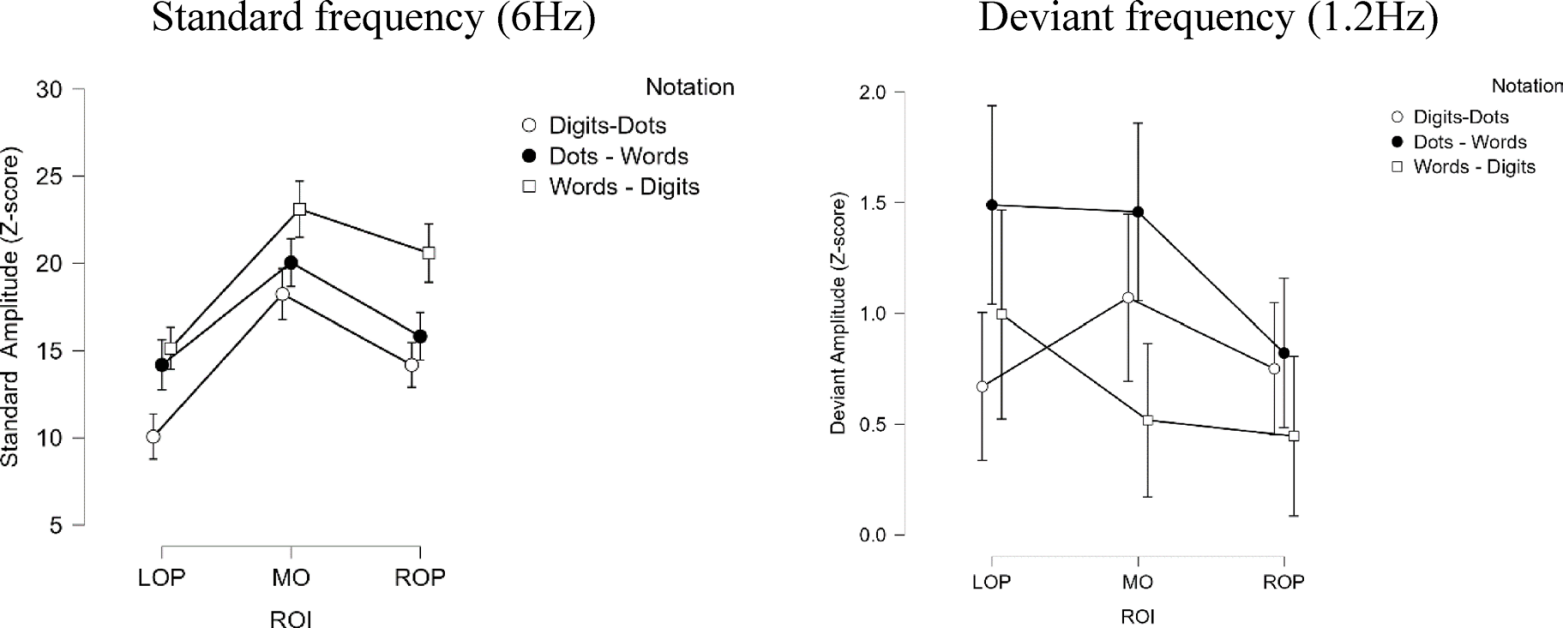
The interaction between Notation and ROI in the Standard (left) and Deviant (right) Frequency Responses. Vertical bars denote 95% Credible Intervals (CI).

Bayesian Repeated Measures ANCOVA (i.e., Bayesian Model Comparison) showed that the model that best explained the data was the model containing the main effect of Notation, the main effect of ROI and their interaction (i.e., Notation + ROI + Notation*ROI), BF_10_ = 7.85 × 10^12^ (see Table 2) This was further corroborated by the comparison of the model-averaged results outlined in Table 3. Here, the BF Inclusion (BF_Incl_) reflects the predictive strength of the effect with respect to the data by comparing all models that include the effect of interest to the models without this effect. The results showed that there is extreme support for the inclusion of the main effect of Notation, BF_Incl_ = 2.96 × 10^6^ and main effect of ROI, BF_Incl_ = 2.76 × 10^6^ and strong support the interaction Notation*ROI, BF_Incl_ = 10.93 (see Fig. 4).

**Table 2:** The outcomes of the Bayesian ANCOVA (i.e., bayesian model comparison—all models) on the standard responses (i.e., stimuli presented at 6 Hz).

Models	*P*(M)	*P*(M|data)	BF_M_	BF_10_	Error%
Null model (incl. subject and random slopes)	0.03	5.65 × 10^− 14^	2.09 × 10^− 12^	1.00	
Notation + ROI + Notation*ROI	0.03	0.44	29.51	7.85 × 10^12^	4.12
Notation + ROI + Age + Notation*ROI	0.03	0.22	10.32	3.86 × 10^12^	4.21
Notation + ROI	0.03	0.09	3.62	1.58 × 10^12^	3.34
Notation + Condition + ROI + Notation*ROI	0.03	0.08	3.32	1.46 × 10^12^	3.73
Notation + Condition + ROI + Age + Notation*ROI	0.03	0.05	1.78	8.15 × 10^11^	8.71
Notation + ROI + Age	0.03	0.04	1.60	7.34 × 10^11^	4.13
Notation + Condition + ROI + Notation*Condition + Notation*ROI	0.03	0.02	0.70	3.28 × 10^11^	4.80
Notation + Condition + ROI	0.03	0.02	0.62	2.92 × 10^11^	3.51
Notation + Condition + ROI + Notation*ROI + Condition*ROI	0.03	9.35 × 10^− 3^	0.35	1.66 × 10^11^	4.22

All models include subject and random slopes for the repeated measures. Only the first ten models out of 38 are depicted.

**Table 3:** Analysis of effects across all models for the general visual response.

Effects	*P*(incl)	*P*(excl)	*P*(incl|data)	*P*(excl|data)	BF_incl_
Notation	0.74	0.26	1.00	1.21 × 10^− 7^	2.96 × 10^6^
Condition	0.74	0.26	0.21	0.79	0.09
ROI	0.74	0.26	1.00	1.29 × 10^− 7^	2.76 × 10^6^
Age	0.50	0.50	0.33	0.67	0.50
Notation*Condition	0.32	0.68	0.04	0.96	0.08
Notation*ROI	0.32	0.68	0.83	0.17	10.93
Condition*ROI	0.32	0.68	0.02	0.98	0.04
Notation*Condition*ROI	0.05	0.95	8.93 × 10^− 5^	1.00	1.61 × 10^− 3^

It is worth noting that in JASP, models that include interactions without the corresponding main effects are excluded because they are considered implausible due to violating the principle of marginality^50,51^. Therefore, to test the model-averaged results for the models including only the interactions, we computed the effects again, but instead of comparing all models, we compared them across matched models^49^. Put differently, models with the interaction effect are compared against all other models, including the same predictors but not the interaction effect and no higher-order interactions. The results of the matched models effect analysis remained similar: there was extreme support for the inclusion of the effect of Notation, BF_Incl_ = 1.35 × 10^6^, of the effect of ROI, BF_Incl_ = 1.15 × 10^6^, and moderate support for the inclusion of the Notation*ROI interaction, BF_Incl_ = 5.12. In sum, a model including only the Notation*ROI, but not the corresponding main effects, demonstrated lower predictive power than the models with the main effects.

Post-hoc comparisons (posterior odds corrected for multiple comparisons, prior odds = 0.59, see Fig. 2S in Supplementary material) for Notation showed that the standard amplitude for the Words – Digits condition was strongest compared to both the Dots – Words (BF_10,U_ = 1.24 × 10^6^, posterior odds = 730369.41) and Digits – Dots conditions (BF_10,U_ = 7.80 × 10^19^, posterior odds = 4.58 × 10^19^). The Dots – Words amplitude was also higher than the Digits – Dots (BF_10,U_ = 32564.39, posterior odds = 419128.36). For the effect of ROI, the amplitudes were the strongest at the MO scalp region compared to both LOP (BF_10,U_ = 7.38 × 10^29^, posterior odds = 4.34 × 10^29^) and ROP (BF_10,U_ = 1.58 × 10^7^, posterior odds = 9.27 × 10^6^). The amplitudes recorded at LOP were the lowest also compared to the ROP, (BF_10,U_ = 2.79 × 10^7^, posterior odds = 1.64 × 10^7^).

To disentangle the interaction between Notation and ROI, we conducted post-hoc Bayesian repeated measures ANOVA per Notation with ROI (3 levels: ROP, LOP, and MO) as a within-subject effect.

For the *Digits – Dots* notation, the Bayesian model comparison showed anecdotal support for the presence of main effect of ROI, BF_10_ = 8.37 × 10^6^. The average z-scores were higher in the MO region, followed by the ROP and the LOP (see Fig. 4S in Supplementary material). This observation was supported by the difference between MO vs. LOP, (BF_10,U_ = 1.01 × 10^6^, posterior odds = 596064.29, prior odds = 0.59), and MO vs. ROP, (BF_10,U_ = 66.21, posterior odds = 38.89, prior odds = 0.59), and the difference between ROP and LOP (BF_10,U_ = 37.45, posterior odds = 22.00, prior odds = 0.59).

For the *Words – Digits* notation, there was again a main effect of ROI, BF_10_ = 21415.91. There was extreme evidence for a difference between MO vs. LOP, (BF_10,U_ = 40398.63, posterior odds = 23730.20, prior odds = 0.59), and MO vs. ROP, (BF_10,U_ = 62.85, posterior odds = 36.92, prior odds = 0.59), but no conclusive evidence for a difference between LOP vs. ROP (BF_10,U_ = 0.60, posterior odds = 0.35, prior odds = 0.59). In sum, the responses to the standard frequency were again stronger in the MO, followed by ROP and LOP.

Finally, for the *Dots – Words* notation, there was also extreme evidence for the presence or a main effect of ROI, BF_10_ = 1269.19. Here, there were differences between MO and LOP, (BF_10,U_ = 2731.30, posterior odds = 1604.37, prior odds = 0.59) and between MO and ROP, (BF_10,U_ = 19.20, posterior odds = 11.28, prior odds = 0.59), but no conclusive evidence for a difference between LOP and ROP, (BF_10,U_ = 0.43, posterior odds = 0.25, prior odds = 0.59)

In sum, the results showed that the general visual response at the standard frequency of stimulation (6 Hz) showed the strongest responses to the Words – Digits, followed by the Dots – Words and Digits – Dots notations. Most importantly for our current hypothesis – there was no evidence for an effect of Condition. Put otherwise, although there were differences in the amplitudes’ strength per Notation and ROI, the general visual response was unchanged across experimental and control conditions. Standard frequency amplitudes were the strongest in the MO region, followed by the ROP and LOR regions. There was also an interaction between the Notation and the ROI factors, suggesting somewhat different amplitude distribution across the ROI depending on the notation. However, for this interaction the assumption of sphericity was violated.

### Response to the deviant frequency (1.2 Hz)

*Assumption checks for the deviant responses* showed that the z-scores per condition were all normally distributed (Shapiro-Wilk *p* > .05), apart from two conditions: Words – Digits and Dots – Words, where the z-score for the experimental conditions in the Medial Occipital ROI showed slight deviations from normality (*Skewness*_words – digits exp MO_ = 0.26, SE = 0.40, *Kurtosis*_words – digits exp MO_ = -1.32, SE = 0.79, *p* = .02 *Skewness*_dots – digits exp MO_ = 1.00, SE = 0.40, *Kurtosis*_dots – digits exp MO_ = 0.72, SE = 0.79, *p* = .02). The residuals are almost all normally distributed (see Fig. 1S in Supplementary Material). Mauchly’s test indicated that the sphericity assumption was violated for the factors of ROI, *p* = .03, and the interactions involving this factor (all *ps* < 0.05).

The Bayesian Repeated Measures ANCOVA (i.e., Bayesian Model Comparison Across All Models) showed that the model that best explained the data was the model containing all the main effects and the interaction between Notation and Condition (i.e., Notation + Condition + ROI + Notation*ROI), BF_10_ = 6.89 × 10^11^ (Table 4) This was further corroborated by the comparison of the model-averaged results outlined in Table 5. Here, the results showed that there is extreme support for the inclusion of the main effect of Notation, BF_Incl_ = 208.77 and main effect of Condition, BF_Incl_ = 8.29 × 10^8^ and strong support for the inclusion of the main effect of ROI, BF_Incl_= 13.82, and the interaction Notation*ROI, BF_Incl_= 13.60 (see Fig. 4).

**Table 4:** Outcomes of the Bayesian ANCOVA (i.e., Bayesian model Comparison – all models) on the deviant responses.

Models	*P*(M)	*P*(M|data)	BF_M_	BF_10_	Error%
Null model (incl. subject and random slopes)	0.03	2.11 × 10^− 13^	7.82 × 10^− 12^	1.00	
Notation + Condition + ROI + Notation * ROI	0.03	0.15	6.30	6.89 × 10^11^	3.16
Notation + Condition + ROI + Notation * ROI + Condition * ROI	0.03	0.13	5.40	6.02 × 10^11^	3.41
Notation + Condition + ROI + Age + Notation * ROI	0.03	0.12	5.27	5.90 × 10^11^	15.30
Notation + Condition + ROI + Age + Notation * Condition + Notation * ROI + Condition * ROI	0.03	0.10	4.03	4.65 × 10^11^	53.24
Notation + Condition + ROI + Age + Notation * ROI + Condition * ROI	0.03	0.08	3.34	3.91 × 10^11^	4.29
Notation + Condition + ROI + Notation * Condition + Notation * ROI	0.03	0.08	3.18	3.74 × 10^11^	3.77
Notation + Condition + ROI + Notation * Condition + Notation * ROI + Condition * ROI	0.03	0.07	2.70	3.22 × 10^11^	4.29
Notation + Condition + ROI + Age + Notation * Condition + Notation * ROI	0.03	0.05	2.04	2.47 × 10^11^	4.93
Notation + Condition + ROI + Notation * Condition + Notation * ROI + Condition * ROI + Notation * Condition * ROI	0.03	0.05	1.93	2.35 × 10^11^	5.40

All models include subject and random slopes for the repeated measures. Only the first 10 models out of 38 are depicted.

**Table 5:** Analysis of effects across all models of the deviant responses.

Effects	*P*(incl)	*P*(excl)	*P*(incl|data)	*P*(excl|data)	BF_incl_
Notation	0.74	0.26	1.00	1.71 × 10^− 3^	208.77
Condition	0.74	0.26	1.00	4.31 × 10^− 10^	8.29 × 10^8^
ROI	0.74	0.26	0.97	0.03	13.82
Age	0.50	0.50	0.45	0.55	0.82
Notation*Condition	0.32	0.68	0.43	0.57	1.61
Notation*ROI	0.32	0.68	0.86	0.14	13.60
Condition*ROI	0.32	0.68	0.51	0.49	2.25
Notation*Condition*ROI	0.05	0.95	0.08	0.92	1.67

The results remained largely similar across the matched models comparison: there was strong support for the inclusion of the effect of Notation, BF_Incl_ = 47.38, extreme support for the inclusion of the effect of Condition, BF_Incl_ = 9.75 × 10^8^, anecdotal support for the inclusion of the effect of ROI, BF_Incl_ = 2.42, and moderate support for the inclusion of the Notation*ROI interaction, BF_Incl_ = 6.58. In sum, a model including only the Notation*ROI, but not the corresponding main effects, demonstrated lower predictive power than the models with the main effects.

Post-hoc comparisons (posterior odds corrected for multiple comparisons, prior odds = 0.59; see Fig. 4S in Supplementary material) for notation showed that the Dots – Words condition differed from both the Digits – Dots (BF_10,U_ = 19.52, posterior odds = 11.46), and Words – Digits (BF_10,U_ = 5183.06, posterior odds = 3044.53) conditions. We observed no evidence for a difference between the Digits – Dots and Words – Digits conditions (BF_10,U_ = 0.21, posterior odds = 0.12). The overall amplitude was highest for the Dots – Words, followed by the Digits – Dots, and finally, Words – Digits. For the Condition, post-hoc comparisons showed extreme support for the difference between experimental and control conditions (BF_10,U_ = 2.09 × 10^32^, posterior odds = 2.09 × 10^32^, prior odds = 1) with the experimental condition yielding higher amplitudes than the control one. Finally, concerning the effect of ROI, post-hoc comparisons (prior odds = 0.59) showed no support for the presence of an overall difference between the MO and LOP regions (BF_10,U_ = 0.08, posterior odds = 0.05), moderate support for the presence of an overall difference between ROP and the LOP (BF_10,U_ = 4.16, posterior odds = 2.44) and strong support for an overall difference between the ROP and MO (BF_10,U_ = 24.66, posterior odds = 14.49). Overall amplitudes were higher for both MO and LOP, compared to the ROP.

Finally, there was an interaction between the Notation and ROI, suggesting a possible differential lateralisation pattern with the responses for Words – Digits and Dots – Words being mostly left lateralised, while for the Digits – Dots, the responses were strongest in the medial ROI. Nonetheless, this interaction should be approached with caution first because the assumption of sphericity was violated and second, because the experimental and control conditions are considered together. However, as depicted in Table 1, no significant z-scores were observed in any of the control conditions.

To disentangle the interaction between Notation and ROI, we conducted post-hoc Bayesian repeated measures ANOVA per Notation with ROI (3 levels: ROP, LOP, and MO) as a within-subject effect (see Fig. 5S in Supplementary material). For the *Digits – Dots* notation, the Bayesian model comparison showed anecdotal support for the presence of main effect of ROI, BF_Incl_ = 1.66. The average z-scores were higher in the MO region, followed by the ROP and the LOP. This observation was moderately supported by the difference between MO vs. LOP, (BF_10,U_ = 4.13, posterior odds = 2.43, prior odds = 0.59). There was anecdotal support for the difference between ROP and MO, (BF_10,U_ = 1.00, posterior odds = 0.58, prior odds = 0.59), and the difference between ROP and LOP was inconclusive (BF_10,U_ = 0.21, posterior odds = 0.12, prior odds = 0.59).

For the *Words – Digits notation*, the Bayesian model comparison showed moderate support for the presence of main effect of ROI, BF_Incl_ = 4.80. The average z-scores were higher in the LOP, followed by the MO and ROP (see Fig. 5 in Supplementary material). This observation was moderately supported by the difference between LOP and MO, (BF_10,U_ = 3.72, posterior odds = 2.19, prior odds = 0.59). There was anecdotal support for the difference between ROP and LOP, (BF_10,U_ = 1.53, posterior odds = 0.90, prior odds = 0.59), and inconclusive for the difference between ROP and MO (BF_10,U_ = 0.20, posterior odds = 0.12, prior odds = 0.59).

For the *Dots - Words* notation, the Bayesian model comparison showed moderate support for the presence of main effect of ROI, BF_Incl_ = 9.67 (see Fig. 5 in Supplementary material). The average z-scores were somewhat similar in LOP and MO, followed by ROP. This observation was moderately supported by the difference between ROP and MO, (BF_10,U_ = 5.17, posterior odds = 3.04, prior odds = 0.59), and ROP and LOP, (BF_10,U_ =3.66, posterior odds = 2.15 prior odds = 0.59). There was inconclusive evidence for the difference between LOP and MO (BF_10,U_ = 0.19, posterior odds = 0.11, prior odds = 0.59).

Taken together these results showed that the deviant frequency responses were strongest for the Dots – Words notation, followed by Words – Digits and finally Digits – Dots notation. There was some evidence that the response was also higher in the left and medial ROI, compared to the right ROI. Most importantly, unlike the results observed in response to the standard stimulation frequency (6 Hz), there was a main effect of condition in response to the deviant frequency (1.2 Hz) with the z-scores of the oddball stimuli being significantly present in the experimental, but not in the control condition.

## Discussion

Mapping between different numerical formats (i.e., digits, number words, and non-symbolic quantities) constitutes and important building block of math competencies^1,4,15,17,18,21–23^. Yet, the neurocognitive mechanisms underlying the relationship between these three numerical formats remain poorly understood. In the current study, we examined whether digits, number words, and dots are automatically integrated in a sample of 7 to 14-year-old children using a frequency-tagged EEG technique.

We used an oddball design during which children passively viewed mixed notation sequences (i.e., digits – dots, words – digits, dots – words). In the experimental condition, these sequences were divided into standard (< 5) and deviant (> 5) magnitude categories and were presented at a fast rate of 6 Hz (i.e., 6 stimuli per second), with every 5th stimulus (i.e., at 1.2 Hz) being a deviant. In the control condition, stimuli of both magnitude categories were randomly intermixed. Based on previous developmental work^21–23^, we expected to observe responses at the deviant frequency 1.2 Hz in the experimental but not in the control conditions. In addition, we expected stronger deviant responses in the dots – words and words – digits, and weaker, if any, in digits – dots condition. Such results would indicate automatic integration of the magnitude information across these formats. We also anticipated notation-dependent lateralisation of the responses, characterised by the presence of an interaction between the notation and region of interest (ROI)^29,34,35,38^. Overall, the results provide evidence for partial representational overlap between digits, number words, and dots.

First, concerning the *general visual response* (i.e., the standard frequency of presentation 6 Hz), we observed evidence for the presence of main effects of notation, region of interest (ROI) and an interaction between these factors. The standard responses were strongest for the words – digits, followed by dots – words and digits – dots. The highest z-scores were recorded at the Medial Occipital (MO) region, followed by the Right-Occipito Parietal (ROP) and finally the Left Occipito-Parietal (LOP). There were also interactions between these two factors, suggesting a notation-depended lateralisation response. However, the lateralisation patterns were not pronounced, and the interaction probably reflects the fact that the amplitudes recorded at the LOP and ROP regions differed only in the dots – digits condition but not in the dots – words and words – digits (see also Figs. 2S and 3S in the Supplementary material). In addition, the assumption of sphericity for this interaction was violated. Therefore, we lack conclusive evidence to interpret this interaction definitively. Finally, and most importantly, there was no evidence for a main effect of the Condition, which indicates that the experimental and control conditions were well matched on their visual properties. Therefore, any significant harmonics in the oddball frequency are unlikely to be due to visual confounds.

In contrast, the main effect of the Condition was present when we analysed the *magnitude-specific responses*. Here, the results showed that the responses to the deviant frequency and its harmonics were present in the experimental but not in the control conditions, although, as it can be seen in Table 1, not all ROIs yielded significant z-scores (i.e., > 1.64). These findings are in line with previous studies using the same EEG-technique in adults and where the authors observed a magnitude discrimination response across digits, number words, and dots^34,35^, and across canonical dot and finger configurations in children^28^. Together, these findings suggesting that numerical formats are automatically integrated under implicit conditions [^30^, but see^13^].

Similarly to the results of the general visual response analyses, we also observed a main effect of Notation. Here, the responses on the deviant frequency across both experimental and control conditions were strongest for the dots – words condition. The response for words – digits and digits – dots were similar in terms of amplitude strength. Finally, there was also some evidence for the presence of a main effect of ROI, yielding highest responses for the MO, followed by ROP and LOP, as well as evidence for an interaction between the ROI and notation. In our post hoc analysis, the evidence for a format-dependent lateralisation pattern was moderate at best and only for words – digits and dots – words. For the words – digits condition, the strongest amplitudes were in the LOP, while for the dots – words both the MO and the LOP showed similar amplitudes. Although the pattern of this interaction is in line with our expectations and previous studies (e.g^34^). These results should be taken with caution since the assumptions of sphericity for the factor ROI as well as the interactions involving this factor were violated. It could be argued that the inconclusive results concerning the interaction effects are due to insufficient data. However, as indicated in the results section, we analysed the data with narrow, wide, and flat priors. This did not lead to any substantial changes in the model comparison and the observed effects, thus showing that the current results are robust.

The current results concerning the main effect of Notation also corroborate to some extent previous developmental behavioral work by Marinova, et al.^21^. In this study, when the authors examined the cross-notation mapping performance of 3-, 4-, and 5- years-olds kindergarten children, it was found that for the 3- and 4-years-olds, the performance was significantly more accurate for the dots – words, followed by the words – digits and dots – digits (see also^22,23^). However, for the older children performance in the words – digits and dots – digits condition was similar. The significant main effect of notation in our current data showed that the overall deviant responses were strongest for dots – words, followed by words – digits and dots – digits, which seems to complement these previous behavioral findings.

Regarding the integration of magnitude-related information across various formats, however, our findings showed only a main effect of Condition, but not an interaction between the Notation and Condition as we initially anticipated. These findings suggest that the numerical integration in primary school children is notation-independent, at least during early processing stages targeted with the fast period stimulation of the present design (i.e., 166 ms). While evidence for an automatic integration across numerical formats, as measured in the current and previous studies^28,34,35^ does not directly imply that the cortical representations for these numerical formats are identical, it is nevertheless in line with the idea that numbers are represented in one common system in school-aged children or at least that there is a partial representational overlap^2–4^. However, it remains possible that at the later stages (> 166 ms), the processing becomes rather format-dependent (e.g^13^). Moreover, in contrast to the present results, our previous study in adults^34^ yielded an interaction between condition and notation. The significant differences between experimental and control conditions for dots – words, and words – digits, but not in dots – digits we observed in adults^34^, suggest that the automatic association between some numerical formats decreases substantially with further development and schooling. In support of this claim, previous neuroimaging studies in adults have demonstrated that a person’s experience and education (as indicated by their arithmetic proficiency) is negatively associated with the degree of representational overlap for digits and dots in the parietal cortex^52^.

Given the relatively wide age range (7–14 years), it is possible that subtle age-related differences in numerical format integration do exist throughout childhood but were not detected in the current study. For instance, a set of fMRI neuroimaging studies demonstrated that children around the age of 11 years, as well as adults recruit largely the same number processing neuronal networks for digits, dots, and number words^11,12,53^. Despite the activation of similar brain regions in children and adults, the functional connectivity of these regions does undergo a substantial change due to maturation mostly related to the domain-general aspects of cognitive control^53^. Finally, a growing body of evidence in both adults^54–57^ and children^58^ suggests that although symbolic and non-symbolic numbers draw on common neuronal substrates, their tunning patterns are format-dependent, thus suggesting distinct underlying neuronal populations. However, definitive conclusions about developmental trajectories of numerical mappings cannot be drawn from the present data. But the descriptively different neuronal response pattern reported in children and adult studies^34^ could be fruitful venues for future studies. In addition, since in the current study the standard stimuli were smaller than 5 and the deviants were larger than 5, it may be worthwhile to test the reverse direction to assess whether the strength of numerical format integration is symmetrical.

Another factor, possibly, influencing the automaticity of numerical format integration—particularly for number words—is the linguistic profile of the children; specifically, whether they are L1 or L2 speakers of the language of math instruction. As outlined in one of our reviews^59^, language background can significantly impact both basic (e.g., counting) and advanced (e.g., arithmetic) numerical skills. Prior evidence in children (e.g^60^), and adults (e.g^61^), further suggests that multilingual individuals may represent symbolic numbers and their lexico-semantic associations differently across languages. These differences may, in turn, influence whether symbolic and non-symbolic numbers are automatically mapped onto one another. Although we included a speeded number word reading task to confirm that children could read the German number words at the same presentation rate as in the frequency-tagged EEG task (i.e., 166 ms), it is important to note that the cognitive demands of these two tasks differ. Thus, potential differences in processing efficiency between L1 and L2 German speakers may also impact the strength of the observed oddball responses. Future studies could adopt similar paradigms to examine whether, and how, the automaticity of cross-format numerical mappings varies by language background, for instance by including number words presented in both L1 and L2, and assessing oddball responses both within and across individuals.

As the research on numerical format processing in children and adults continues, it is worth considering an additional line of research suggesting that both estrangement and integration are possible depending on the methodological choices and the underlying stimulus and task characteristics. Concretely, studies have demonstrated that the evidence for shared versus format-dependent representations also depends on methodological factors such as task instructions and even the use of single vs. mixed formats sequences, as well as the order of stimulus presentation^31^ but see^62,63^. For instance, behavioural and neurocognitive studies in adults suggest that cross-format integration or estrangement depends on task requirements with evidence for the estrangement being present in explicit (e.g., number comparison) condition, while integration is observed under implicit instructions (e.g^31^. On one side, behavioural studies using explicit task instructions that require processing the magnitude of numbers (e.g., number comparison, numerical matching task, match-to-sample task, etc.) have mostly found evidence for estrangement^10,13,31,62–64^. On the other, under implicit task instructions (e.g., letter-number discrimination task), previous research have reported evidence for an integration^28,30,31,34^. This means that, we may observe response pattern in line with integration or estrangement depending on the task due to top-down modulations^29^. For instance, goal-directed task instructions such as number comparison encourage participants to employ more cognitive resources. This leads to a finer tuning of the activated numerical representations, resulting in format-dependent performance. In addition, explicit task instructions require participants to retain the numerical stimuli in their working memory, thus potentially inhibiting the associative strength between symbolic and non-symbolic numbers^31^. Implicit task instructions on the other hand, may lead to broader neuronal activation, in which case there will be more overlap between the numerical format representations.

Another methodological factor influencing number representation may be the choice of presenting mixed or single format conditions. Similarly to previous studies employing within vs. cross-format conditions^10,31,62^, Fu and colleagues^65^ obtained different ERPs in response to numerical quantity depending on whether the block was within (dots - dots) or cross-format (dots - digits), with the latter condition eliciting overall larger response amplitudes. Drawing on working-memory literature, the authors propose that presenting numerical information in cross-format conditions could lead to a more precise coding of the underlying representation. Although it is often assumed that mixing across formats, will encourage participants to rely on an abstract numerical representation^62,66,67^, pinning formats against each other may also potentially increase the format-specific discrimination abilities. Future research should examine how instructions and presentation formats influences the numerical format integration across development.

In sum, in the current study we used an oddball frequency-tagged EEG methodology to examine whether primary school children (7 to 14 years old) automatically extract magnitude-related information across numerical formats (digits, words, and dots). In line with previous developmental studies, the results showed that overall deviant responses were strongest for dots – words, followed by words – digits and digits – dots. The presence of significant deviant responses in the experimental condition but not in the control condition, suggested that magnitude-related information is extracted independently of the format, at least under implicit and rapid representation conditions. Overall, these findings suggest that symbolic and non-symbolic numerical representations may partially overlap in school-aged children.

## Electronic supplementary material

Below is the link to the electronic supplementary material.

Supplementary Material 1

## Data Availability

The study’s data set and statistical output are freely available on the Open Science Framework (https://osf.io/ktm94/).
