# Supplementary material for: Numerical format integration in primary school children examined with frequency-tagged electroencephalography
Source: Sci Rep. 2025 Aug 5;15:28641. doi: 10.1038/s41598-025-11281-7 (PMC12325568; doi:10.1038/s41598-025-11281-7)
Supplement: Supplementary file 1 — Supplementary Material 1 [file 41598_2025_11281_MOESM1_ESM.docx]

**Numerical Format Integration in Primary School Children Examined with Frequency-Tagged Electroencephalography**

**SUPPLEMENTARY MATERIAL**

Mila Marinova^1,2, 3^ & Christine Schiltz^1^

^1^Institute of Cognitive Science and Assessment, Department of Behavioural and Cognitive Sciences, Faculty of Humanities, Education and Social Sciences, University of Luxembourg, Esch-Belval, Luxembourg.

^2^Brain and Cognition, KU Leuven, Leuven, Belgium

^3^Faculty of Psychology and Educational Sciences, KU Leuven @Kulak, Kortrijk Belgium.

Correspondence concerning this article should be addressed to Mila Marinova, Institute of Cognitive Science and Assessment, Department of Behavioural and Cognitive Sciences, Faculty of Humanities, Education and Social Sciences, University of Luxembourg, Maison des Sciences Humaines 11, Porte des Sciences; L-4366 Esch-sur-Alzette Esch-Belval, Luxembourg. Tel: +32468219730; E-mail: mila.g.marinova@gmail.com

ORCID :

Mila Marinova: <https://orcid.org/0000-0002-6875-7742>

Christine Schiltz: <https://orcid.org/0000-0002-1055-3878>

| Standard Frequency Responses | Deviant Frequency Responses |
| --- | --- |
| 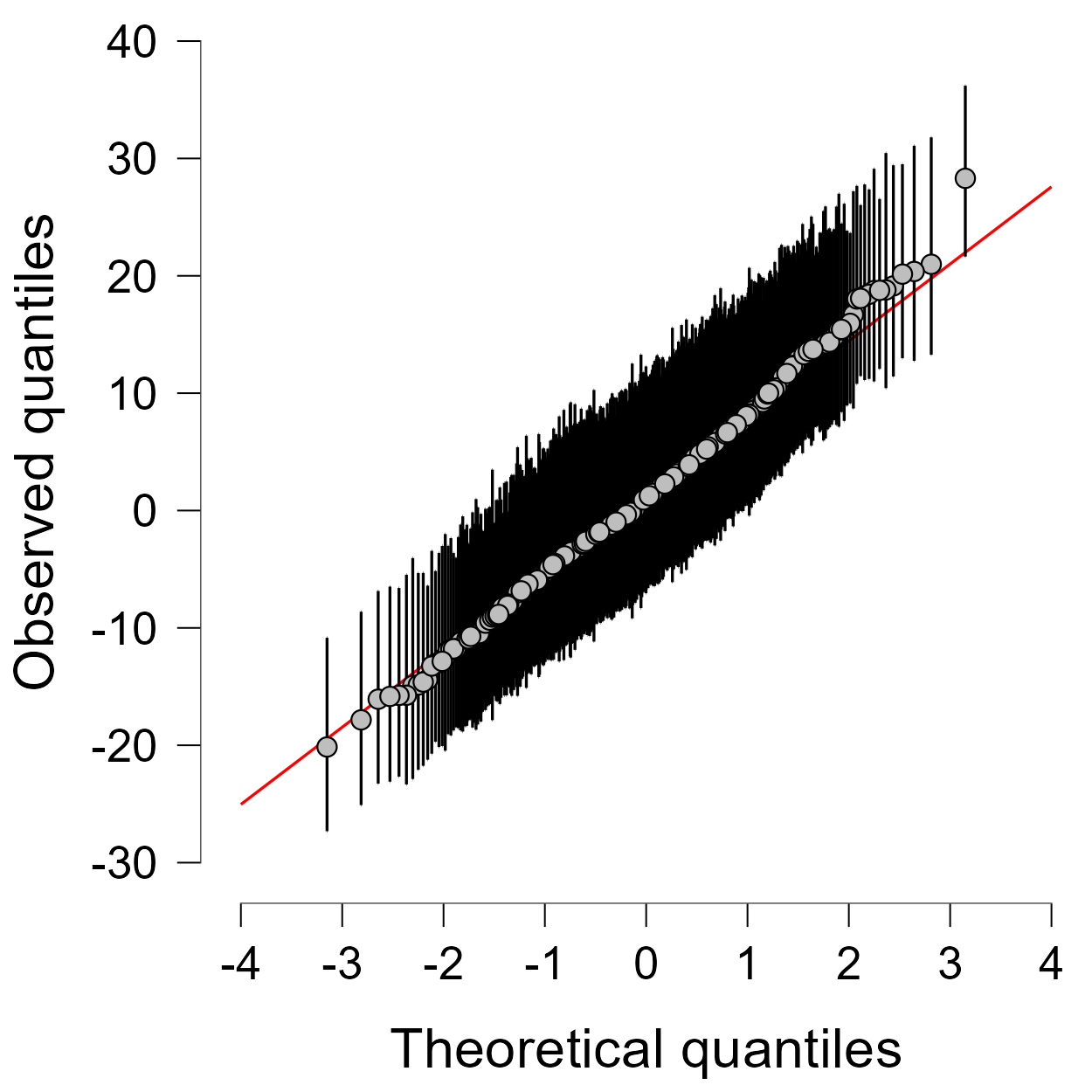 | 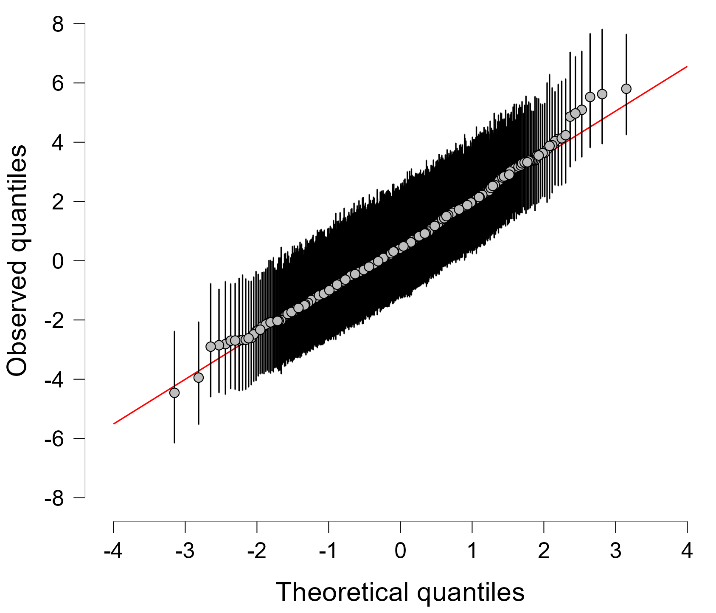 |

Figure 1S. Q-Q plot visualising the observed residuals against the residuals of a standard normal distribution for the standard and deviant frequencies.

Note 1. The data is perfectly normally distributed if all the points are on the red line. The vertical bars through each point represent 95% Central Credible Interval.

*
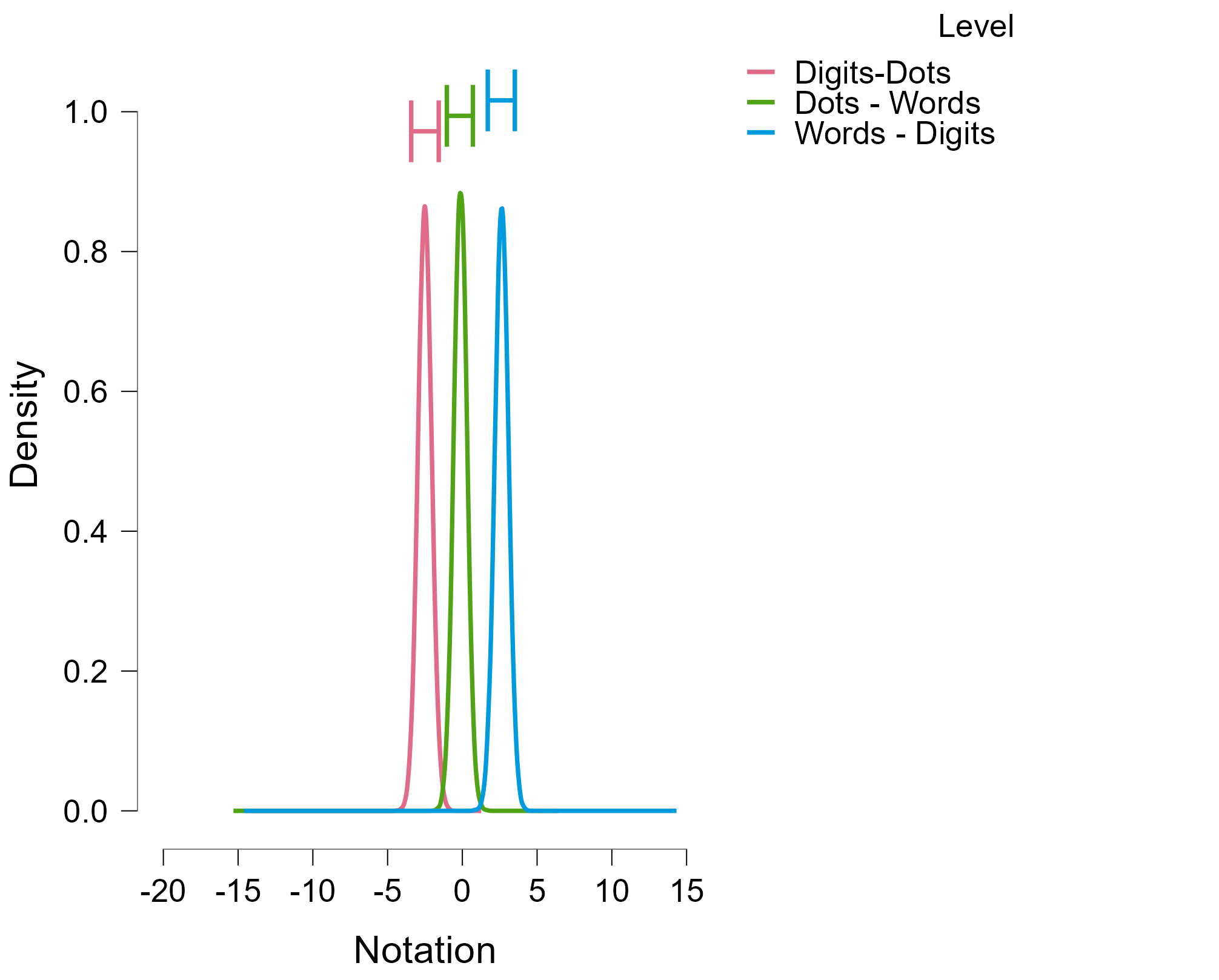

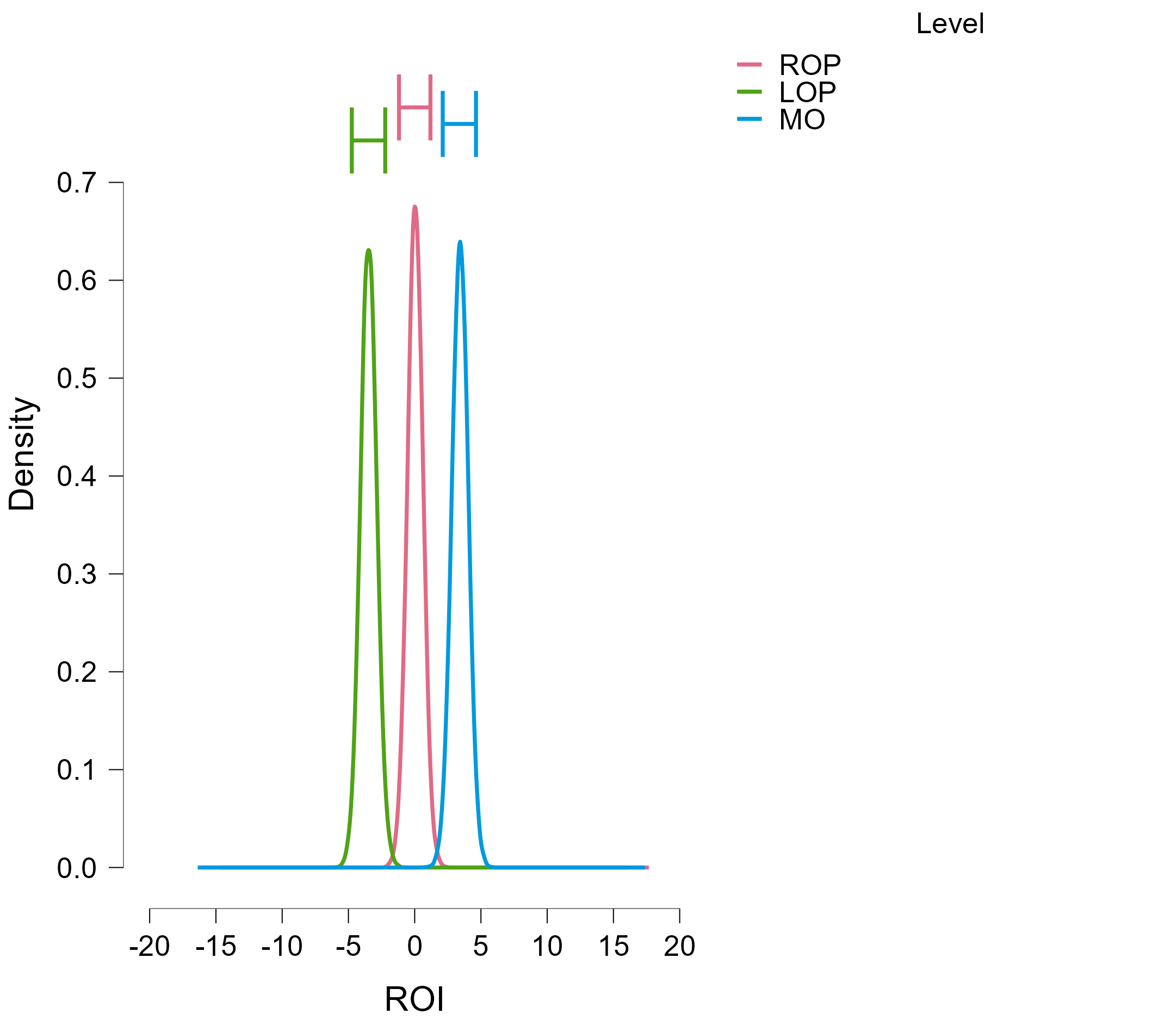
*
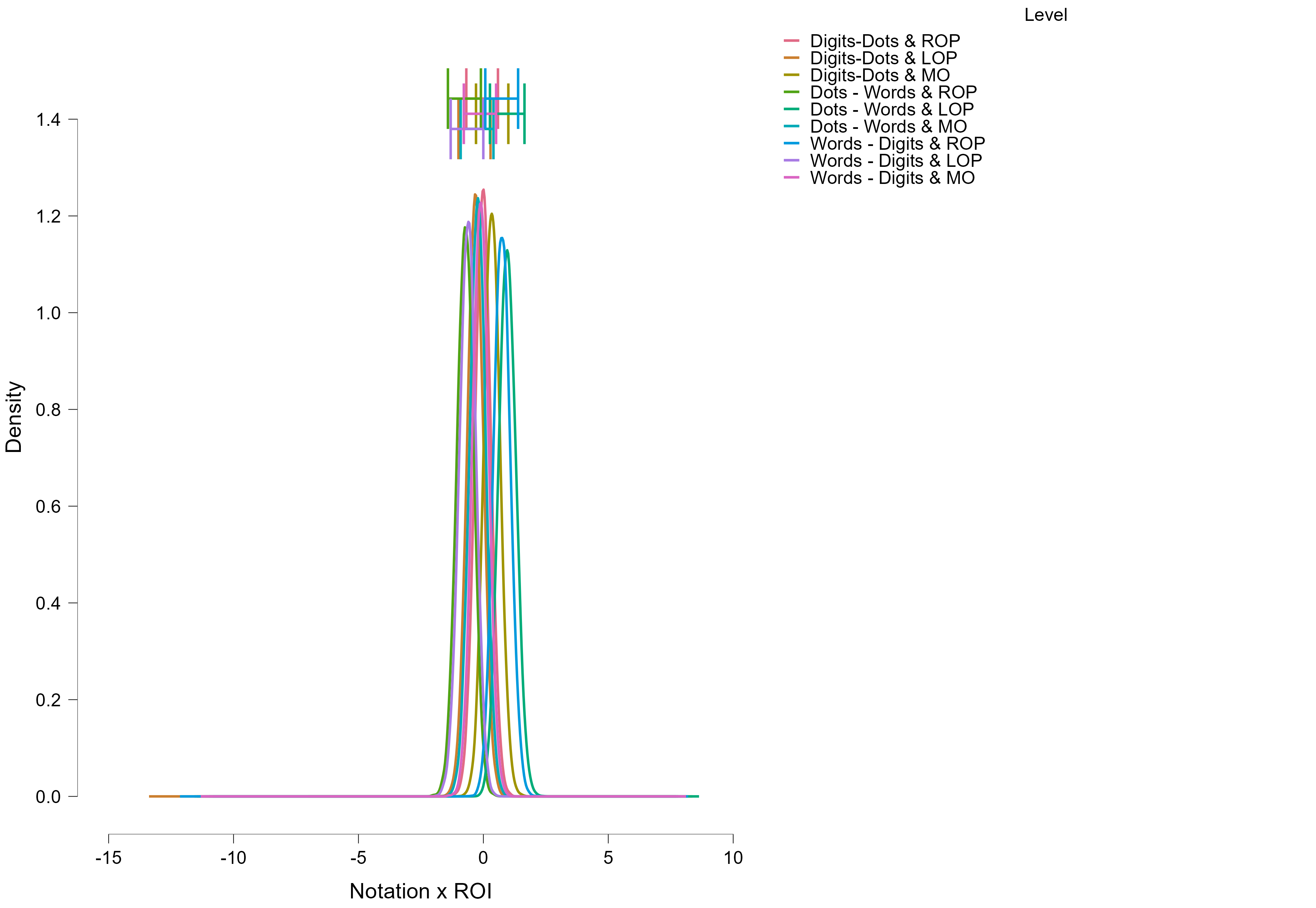


Figure 2S. Posterior distributions of the effect of each included factor and their interaction on the strength of the standard amplitude. Horizontal bars above each density denote 95% Credible Intervals (CI).

**Note 2**. ROP = Right Occipito Parietal; LOP = Left Occipito Parietal; MO = Medial Occipital

**Figure 3S. Model averaged posterior distributions for the effect of Region of Interest (ROI) on the overall strength of the standard amplitude averaged across experimental and control conditions for each notation. Horizontal bars** above each density **denote 95% CI.**

**Note 3**. ROP = Right Occipito Parietal; LOP = Left Occipito Parietal; MO = Medial Occipital

Figure 4S. Posterior distributions of the effect of each included factor and their interaction on the strength of the deviant amplitude. Horizontal bars above each density denote 95% Credible Intervals (CI).

**Note 4**. ROP = Right Occipito Parietal; LOP = Left Occipito Parietal; MO = Medial Occipital

Figure 5S.Model averaged posterior distributions for the effect of Region of Interest (ROI) on the overall strength of the deviant amplitude averaged across experimental and control conditions for each notation. Horizontal bars above each density denoted 95% CI.

Note 5. ROP = Right Occipito Parietal; LOP = Left Occipito Parietal; MO = Medial Occipital
